# Differential Engraftment of Parental A20 PD-L1 WT and PD-L1 KO Leukemia Cells in Semiallogeneic Recipients in the Context of PD-L1/PD-1 Interaction and NK Cell-Mediated Hybrid Resistance

**DOI:** 10.3389/fimmu.2022.887348

**Published:** 2022-06-20

**Authors:** Maria-Luisa del Rio, Jose-Antonio Perez-Simon, Jose-Ignacio Rodriguez-Barbosa

**Affiliations:** ^1^ Transplantation Immunobiology and Immunotherapy Section, Institute of Molecular Biology, University of Leon, Leon, Spain; ^2^ CIBERONC Consortium, Accion Estrategica en Salud, Spain; ^3^ Department of Hematology, University Hospital Virgen del Rocio/Institute of Biomedicine [Instituto de Biomedicina de Sevilla (IBIS)/Centro Superior de Investigaciones Científicas (CSIC)/Centro de Investigación Biomédica en Red Cáncer (CIBERONC)], Seville, Spain

**Keywords:** PD-1, CD80, PD-L1, PD-L2, NK cells, hybrid resistance, A20 leukemia cells

## Abstract

The contribution of natural killer (NK) cells to tumor rejection in the context of programmed death-ligand 1/programmed death 1 (PD-L1/PD-1) blockade is a matter of intense debate. To elucidate the role of PD-L1 expression on tumor cells and the functional consequences of engaging PD-1 receptor on cytotoxic cells, PD-L1 expression was genetically inactivated and WT or PD-L1-deficient parental tumor cells were adoptively transferred intravenously into F1 recipients. The engraftment of PD-L1-deficient A20 tumor cells in the spleen and liver of F1 recipients was impaired compared with A20 PD-L1 WT tumor counterparts. To elucidate the mechanism responsible for this differential tumor engraftment and determine the relevance of the role of the PD-L1/PD-1 pathway in the interplay of tumor cells/NK cells, a short-term competitive tumor implantation assay in the peritoneal cavity of semiallogeneic F1 recipients was designed. The results presented herein showed that NK cells killed target tumor cells with similar efficiency regardless of PD-L1 expression, whereas PD-L1 expression on A20 tumor cells conferred significant tumor protection against rejection by CD8 T cells confirming the role of the co-inhibitory receptor PD-1 in the modulation of their cytotoxic activity. In summary, PD-L1 expression on A20 leukemia tumor cells modulates CD8 T-cell-mediated responses to tumor-specific antigens but does not contribute to inhibit NK cell-mediated hybrid resistance, which correlates with the inability to detect PD-1 expression on NK cells neither under steady-state conditions nor under inflammatory conditions.

## Introduction

Bone marrow-derived natural killer (NK) cells are a population of innate type I lymphoid cells (ILC-1) essential during the early phase of antiviral responses for the contention of viral spread. NK cells efficiently kill tumor cells and eliminate stressed cells without relying on major histocompatibility complex (MHC) specificity. Kärre and Ljunggren introduced the theoretical framework of the *missing self-concept* that accounted for the observations of hybrid resistance and the rejection of tumor cells that had low or had lost expression of MHC class I molecules (1). While T-cell cytotoxic responses depend on MHC restriction, NK cell recognition of non-self, as it lacks TcR, depends on a balance of positive (activating) and negative (inhibitory) receptor signals received from co-stimulatory and co-inhibitory ligands expressed in stressed cells or tumor cells when exposed to proinflammatory cytokines (2). A misbalance of these dominant co-inhibitory ligands on the target cells may occur for instance due to modifications of self-MHC class I molecule expression that would trigger NK cell cytotoxicity. Killer cell immunoglobulin-like receptors (KIRs) in humans and Ly49 in mice and NKG2A in both species are the most relevant dominant MHC class I-dependent co-inhibitory pathways. Apart from these classical regulators of NK cell function, NK cells may also depend on the recognition of other non-MHC class I inhibitory receptors, also known as immune inhibitory checkpoints [programmed death 1 (PD-1), BTLA, CD160, TIGIT, etc.], which are poorly characterized so far (3, 4).

NK cell function was first described in the 1960s, as the effector cells responsible for mediating *hybrid resistance* to parental bone marrow transplantation in lethally irradiated semiallogeneic F1 recipients (5–9). In addition to NK cells, CD8 T cells can also recognize hematopoietic antigens and tumor-specific antigens in parental tumor cells and contribute to resist the engraftment of parental cells, although to a lesser extent (10, 11).

The discovery of PD-1 as a receptor capable of conveying negative signals to T cells (12, 13) and, soon after, the therapeutic implications of the programmed death-ligand 1 (PD-L1)/PD-1 immune checkpoint blockade brought great excitement to the field of cancer immunotherapy (12, 14). Most of the antitumor cytotoxic activity achieved after PD-L1/PD-1 blockade has been assigned to enhance CTL responses (15–17; PD-L1 et al., 2018). Despite this claim, some authors have attributed the antitumor properties to NK cells or even macrophages in the context of PD-L1/PD-1 therapeutic blockade (18–26).

The motivation of this study was to elucidate whether or not the co-inhibitory receptor PD-1 was involved in the functional activity of NK cells. The aim was to bring some insight into the controversy derived from the difficulty of detecting PD-1 expression in human and mouse NK cells under homeostatic or inflammatory conditions (21–26). Bearing that in mind, we designed an experimental approach in which parental PD-L1 WT or PD-L1-deficient A20 leukemia cells were injected intravenously or intraperitoneally into semiallogeneic F1 recipients to study the role of PD-L1 expression on tumor cells in NK cell-mediated rejection and to assess the putative involvement of PD-1 co-inhibition in hybrid resistance to tumor implantation. We confirmed that NK cells and to lesser extent host CD8 T cells contributed to the phenomenon of hybrid resistance in the context of parental tumor cell engraftment into semiallogeneic F1 recipients. The expression of PD-L1 on tumor cells diminished tumor rejection by CD8 T cells but did not influence NK cell-mediated rejection, as they were capable of eliminating PD-L1 WT and KO tumor cells with similar efficiency, arguing against the claim that the co-inhibitory receptor PD-1 would play an inhibitory role in NK cell-mediated antitumor responses.

## Material and Methods

### Animal Source

#### Mice

C57BL/6J mice were purchased from Janvier (France). Eight- to 12-week-old female F1 hybrid mice (Balb/c AnN × C57BL/6J) (H-2^d/b^) were bred at the animal facility of the University of Leon for internal use in our experiments. All animals were maintained with a 12-h dark–light cycle at 22°C temperature and received *ad libitum* food and water.

The Animal Welfare Committee of the University of Alcala de Henares (Madrid) in accordance with the European Guidelines for Animal Care and Use of Laboratory Animals approved all experiments with rodents (authorization # OH-UAH-2016/015).

### Hybridoma Cell Lines and Purification of Depleting Antibodies for *In-Vivo* Use

Hybridoma cell lines secreting anti-mouse NK1.1 antibody (clone PK136, mouse IgG_2a_, kappa light chain) and anti-mouse CD8 antibody (clone 2.43, rat IgG_2b_, kappa light chain) or purified isotype-matched controls (anti-CD45.1, clone A20, mouse IgG_2a_, k, in-house made and clone RTK4530, rat IgG_2b_, k Biolegend, San Diego, California) were initially grown in Petri dishes to permit their expansion. Cell lines were gradually adapted to grow in serum-free medium (SFM) (Thermo Fisher Scientific, Waltham, Massachusetts, United States) supplemented with 0.25% of IgG-depleted fetal calf serum (FCS) and then scaled up to spinner flasks of 3-L volume. The cell culture supernatants were centrifuged, prefiltered, and purified by protein G Sepharose affinity chromatography. The eluted fraction of the purified antibodies was dialyzed against phosphate-buffered saline (PBS), and finally, the purified antibodies were passed through a 0.22-μm filter. The purified antibodies for *in-vivo* use were stored frozen at −80°C in endotoxin-free PBS at a concentration of 1–5 mg/ml containing less than 2 EU/ml of endotoxin [Pierce (Thermofisher brand company, Waltham, Massachusetts, USA)].

### A20 Lymphoma Tumor Cell Line

The A20 transplantable leukemia cell line was derived from B lymphocytes of a naturally occurring reticulum cell sarcoma from an old Balb/c AnN mouse (H-2^d^, TIB-208, ATCC, American Type Culture Collection, Manassas, VA, USA) (27, 28). Cells were grown in complete RPMI-1640 medium (Sigma-Aldrich, St. Louis, MO, USA) supplemented with 10% fetal calf serum (Hyclone, Logan, Utah, USA), 2 mM L-glutamine (Sigma), 1 mM pyruvate, (Sigma), non-essential amino acids, and 0.05 mM 2-mercaptoethanol (Sigma-Aldrich, St. Louis, MO, USA) at 37°C and 5% CO_2_. The A20 cell line and its derivatives were routinely tested by PCR to rule out the presence of mycoplasma contamination.

### CRISPR–Cas9-Mediated Generation of PD-L1-Deficient A20 Leukemia Cells

PD-L1 expression in the A20 cell line was knocked out by CRISPR–Cas9 (Clustered, regularly interspaced, short palindromic repeats–associated nuclease Cas9) technology (29, 30). pLenti-CRISPR-V2 plasmid encoding Cas9 and a puromycin resistance cassette (Addgene #52961) was used to clone an oligo DNA guide that was previously validated for the introduction of indel mutations into the PD-L1 gene (15, 31).

The A20 tumor cell line was then transduced with lentiviral particles produced in HEK293T cells, co-transfected with second-generation packaging plasmid psPAX2 (Addgene #12260) and envelope pCMV-VSV-G (Addgene #8454) along with the targeting vector pLenti-CRISPR-V2 plasmid encoding Cas9, puromycin cassette, and oligo DNA guide for exon 3 (Addgene #52961). PD-L1-deficient clones or emptied plasmid-transduced A20 PD-L1 WT tumor cells were selected in the presence of 1 μg/ml puromycin and cloned by limiting dilution. Several PD-L1-deficient cell lines were derived and screened by flow cytometry using an anti-PD-L1 monoclonal antibody (clone MIH5, rat IgG_2a_) (32). To characterize the mutation introduced within exon 3, a set of flanking primers was designed to amplify the mutated gene, and the PCR product was later sequenced at the core DNA sequencing facility of the University of Leon. The lack of protein expression on the surface of the tumor cells was checked by flow cytometry. The sequence of mouse PD-L1 mutation in A20 PD-L1-deficient tumor cells was deposited in GenBank under the accession number OM975989.

### Systemic Parental A20 Tumor Implantation Into Semiallogeneic F1 Recipients

The optimal number of A20 tumor cells to achieve their engraftment in F1 recipients capable of overcoming hybrid resistance was titrated after intravenous injection of distinct cell numbers. The number of 5 × 10^6^ tumor cells was chosen for the *in-vivo* experiments based on the kinetics of tumor implantation and dissemination in F1 recipients. This number of tumor cells reached a similar engraftment level to that of injection of 1 × 10^6^ of A20 tumor cells in syngeneic Balb/c mice, 1 month after the adoptive transfer. However, as expected, the extent of tumor engraftment was reduced in the liver, spleen, and bone marrow of F1 recipients when compared to the syngeneic setting due to the hybrid resistance mechanisms active in the former and absent in the latter (data not shown).

A20 PD-L1 WT and A20 PD-L1-deficient leukemia tumor cells were grown and expanded in a culture medium at a cell density of 3 × 10^5^ cells/ml, collected at the logarithmic phase of cell growth, washed and resuspended at 5 × 10^6^ cells in 200 μl of PBS, and injected i.v. with a 25-G needle. Eight- to 12-week-old semiallogeneic F1 female mice were injected intravenously with either A20 WT or A20 PD-L1 KO tumor cell lines and were euthanized a month after the adoptive transfer of the tumor cells.

### A20 Leukemia Mouse Model of Tumor Implantation Into the Peritoneal Cavity

We adapted a previously reported peritoneal cavity model of tumor implantation for the assessment of short-term A20 tumor survival (33). The cells (5 × 10^6^) of each tumor cell line, either A20 PD-L1 WT or PD-L1 KO, were co-injected intraperitoneally, and 6 days later, the remaining tumor cells within the cavity were harvested by peritoneal lavage and stained with an antibody panel that allowed us to distinguish tumor cells (K^d+^/K^b−^) from host F1 cells (K^d+^/K^b+^). Within the gate of tumor cells, the use of anti-PD-L1 antibody staining differentiates PD-L1 WT from PD-L1 KO A20 tumor cells. To determine the contribution of NK cells or CD8 T cells to hybrid resistance of parental tumor implantation, these immune cells were exhaustively depleted by injection of anti-NK1.1 antibody (clone PK136) or anti-CD8 T cell antibody (clone 2.43), respectively. Two milligrams of antibody/mouse/dose was injected i.p. at day −5 and day −1 prior to the co-injection of A20 WT and A20 PD-L1 KO leukemia cell lines.

For the harvest of tumor cells remaining in the peritoneal cavity, mice were injected with 6 ml of macrophage buffer composed of Dulbecco’s PBS (Ca/Mg free) (Gibco, Thermofisher company brand, Waltham, Massachusetts, USA 14200-067), tetrasodium EDTA (Sigma, E-6511) (0.02%, 0.53 mM), glucose (Sigma, G-7528) (0.1%), and gentamycin (50 μg/ml). The lavage solution was left inside the peritoneal cavity for 2 min and then was harvested with a Pasteur pipette. The volume collected from each mouse was variable and lower than the volume injected. Then, the values obtained were normalized to the volume injected in order to calculate the absolute cell number in the peritoneal cavity (tumor and non-tumor cells) (34).

### Flow Cytometry for the Immunophenotyping of Immune Cells in Primary and Secondary Lymphoid Organs, Peritoneal Cavity, and Tumor-Infiltrating Leukocytes in Metastatic Hepatic Lesions

To distinguish tumor cells from non-tumor cells (host F1 cells) in different hematopoietic compartments and in tumor metastasis of the liver, cellular suspensions were prepared and stained with specific antibodies against MHC class I allele K^b^ (clone AF6-88.5) and MHC class I allele K^d^ (clone SF1-1.1).

**Table 1** shows the list of biotinylated- or fluorochrome-labeled antibodies against cell surface markers that were used to monitor protein expression on the surface of tumor cells and immune cells located in primary and secondary lymphoid organs and tumor-infiltrating cells of the liver metastases. Biotinylated antibodies were developed with streptavidin (SA)–PE, SA–PECy7, or SA–BV421. All these antibodies were purchased from Biolegend or were produced, labeled, and titrated in our own laboratory. Fc receptors were blocked by incubating cell suspensions with 2 μg/ml (0.2 μg/1 × 10^6^ cells) of homemade blocking anti-FcγR mAb (2.4G2) to reduce non-specific binding before adding the abovementioned mAbs (35). Dead cells and debris were systematically excluded from the acquisition gate by adding propidium iodide (PI) at the end of the staining, prior to data acquisition. Living cells were gated as PI negative and aggregates were gated out based on the FSC-H/FSC-A dot plot profile. Flow cytometry acquisition was conducted on a Beckman Coulter CyAn 9 flow cytometer or on a Cytek^®^ Aurora Spectral Cytometer and data analysis was performed using FlowJo software version 10.

**Table 1:** List of antibodies describing the specificity, labeling, clone name and the provider.

Receptor	Label	Clone	Company
CD3	BV711	17A2	Biolegend, (#100241)
Kd	FITC	SF1-1.1	Biolegend, #116606
Kb	Alexa Fluor 647	AF6-88.5	Biolegend, # 116512
CD49b	APC	DX5	Biolegend, #108910
PD-1	BV421	29F.1A12	Biolegend, #135217
PD-L1	Bio	MIH5	Home-made, (74, 32)
NKp46	Bio	29A1.4	Biolegend, # 137616
CD8	PE	53-6.7	Biolegend, # 100708
CD11b	PerCP-Cy5.5	M1/70	BD, #561114
Ly6C	Bio	Monts-1	Home-made (72)
Ly6G	PE	1A8	BD, # 551461
CD4	PE-Cy7	GK1.5	Biolegend, # 100422
B220	Bio	RA3-6B2	Biolegend, # 103203
B220	FITC	RA3-6B2	Thermofisher, # 48-0452-82
PD-L2	Bio	TY25	Thermofisher, #13-5986-85
CD80	Bio	16-10A1	Thermofisher, # 13-0801-81
Ki-67	Bio	SolA15	eBioscience 13-5698-82
Isotype control mIgG2b	Bio	MPC-11	Home-made (71)
Isotype control rat IgG2a	Bio	AFRC MAC 157 (ECACC)	Home-made (70)

List of antibodies describing the specificity, labeling, clone name and the provider.

### Statistical Analysis

Unpaired Student’s *t*-test and two-way ANOVA and a post-analysis based on Tukey’s test were applied to compare the differences of means between the PD-L1 WT and PD-L1 KO tumor groups. These statistical analyses were performed under the conditions of independence of the data, normality test (Kolmogorov test), and equal variances among groups (Bartlett’s test). The statistical analysis was performed using GraphPad Prism 7.0 software (GraphPad Software, Inc., San Diego, CA, USA). A value of *p <*0.05 was considered statistically significant.

## Results

### CRISPR/Cas9 Gene Inactivation of PD-L1 Expression in A20 Leukemia Cells

To evaluate the *in-vivo* role of PD-L1 expression on tumor cells, the CRISPR/Cas9 approach was applied for the genetic introduction of indel mutations into the PD-L1 encoding gene expressed on the surface of the A20 leukemia cell line to abolish protein expression (30, 31). The sequence-encoding mouse PD-L1 was retrieved from the NCBI database with accession number NM_021893.3 to design the targeting strategy to functionally inactivate exon 3 that encodes 2 bp of the signal peptide and the complete Ig V-like domain. We took advantage of a sgRNA guide previously validated in a different tumor model (EG7-OVA cell line derived from T-cell lymphoma EL-4) (15). This sgRNA guide targeted a sequence located at the proximal exon 3 encoding the Ig V extracellular domain of the PD-L1 molecule. The indel mutations introduced into the PD-L1 gene were PCR-amplified and the amplicon was characterized by gene sequencing. The expected band for PD-L1 exon 3 in the A20 PD-L1 WT cell line was 342 bp, whereas in the PD-L1-deficient cell line, it was 326 bp. The indel mutation consisted of an insertion of 5 bp after the codon encoding amino acid Arg (R, position 84) and a deletion of 21 bp from Ala (A, position 85) to Gln (Q, position 91) within exon 3, leading to a frameshift mutation and the introduction of several stop codons (**Supplementary Figures 1A–C**).

### A20 Leukemia Tumor Cells Express *In-Vivo* PD-L1, Whereas CD80 Was Barely Expressed and PD-1 and PD-L2 Were Undetectable

**Figure 1:**
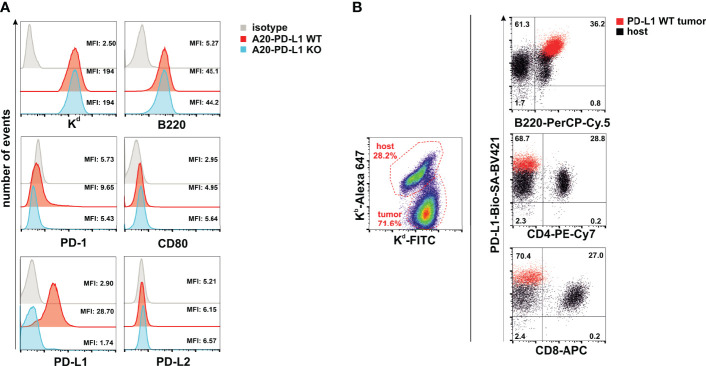
A20 leukemia cell line is negative for programmed death 1 (PD-1) and programmed death-ligand 2 (PD-L2), but barely expressed CD80, leaving programmed death-ligand 1 (PD-L1) freely available to interact in *trans* with PD-1 expressed in immune cells. **(A)** Upper panel: Flow cytometry histograms displaying the staining with the isotype control and anti-K^d^ (H-2^d^) and anti-B220 antibodies in A20 PD-L1 wild-type (WT) and A20 PD-L1 knockout (KO) leukemia cells. Middle and lower panels: Flow cytometry histograms showing the pattern of expression of PD-1, CD80, PD-L1, and PD-L2 in A20 PD-L1 WT compared to KO tumor cells. Notice that in A20 PD-L1-deficient cells, PD-L1 protein expression is absent on the cell surface, whereas the level of expression of the other molecules of the pathway is similar to that observed in A20 PD-L1 WT leukemia cells. This experiment was repeated three times with similar results. A representative histogram displaying the mean fluorescence intensity (MFI) value for each biomarker of the PD-L1/PD-L2/CD80/PD-1 pathway is shown along with the expression of MHC class I (K^b^) and B220. **(B)** Overlapped dot plot illustrating simultaneously PD-L1 expression on host B cells (B220-positive cells), host CD4 T cells, and host CD8 T cells (black dots) infiltrating the tumor and on A20 tumor cells (red dots) in metastatic nodules of the liver of F1 recipients collected at day 30 after the adoptive transfer of tumor cells.

PD-L1 is the ligand of two members of the immunoglobulin superfamily (PD-1 and CD80), and binding to these two receptors in *trans* delivers co-inhibitory signals that co-inhibit T-cell function (12, 36). The PD-L1/PD-1/CD80 and PD-L2/PD-1 pathways represent an example of multiple receptor–ligand interactions in which *trans* interplay with nearby cells is likely to be conditioned by co-expression of paired molecules on the same cell (*cis* interaction) (37). Thus, the co-expression of CD80 and PD-L1 in *cis* on tumor cells prevents PD-L1 from the tumor to deliver co-inhibitory signals in *trans* to T cells (38, 39). This occurs because PD-L1/CD80 *cis* heterodimerization inhibits both PD-L1/PD-1 and CD80/CTLA-4 interactions but maintains the ability of CD80 to activate T cells through the co-stimulatory receptor CD28 (40). The A20 leukemia transplantable cell line was chosen as the tumor model because PD-1 and PD-L2 cell surface receptors are completely absent and the expression of CD80 is barely detectable, whereas PD-L1 is clearly expressed (**Figure 1A**). The *in-vivo* expression of PD-L1 on A20 WT tumor cells (red dots) present in the metastatic nodules of the liver is higher than on either host B cells, CD4 T cells, or CD8 T cells (black dots) (**Figure 1B**). We postulated that in this tumor mouse model, PD-L1 co-inhibitory function would not be compromised by CD80 interaction in *cis* due to its weak expression, allowing PD-L1 freely to engage PD-1 inhibitory receptors that might be present on NK cells and modulate their functional responses.

In summary, the A20 leukemia transplantable cell line is a convenient tumor model for the assessment of the contribution of PD-L1 expression on hematopoietic tumor cells without the interference of CD80 co-expression, which permits the interplay of tumor PD-L1 with PD-1 expressed in immune cells to inhibit their function.

### *In-Vitro* Tumor Cell Growth Rate Was Not Compromised in PD-L1-Deficient Tumor Cells

**Figure 2:**
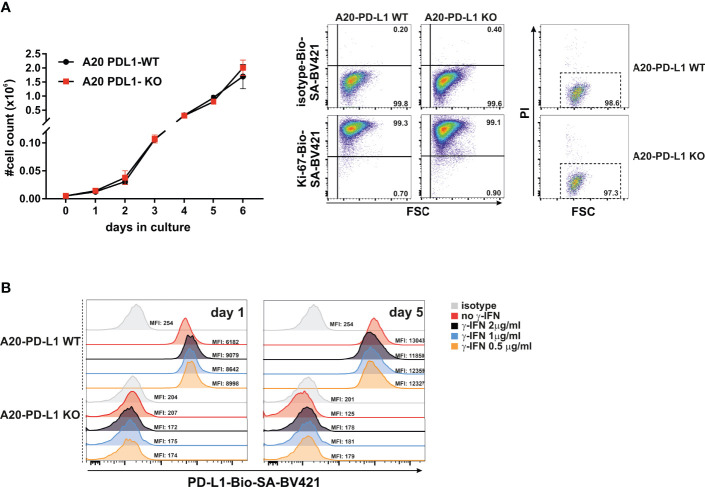
Similar *in-vitro* proliferation rate of A20 PD-L1 WT and PD-L1 KO leukemia cells and *in-vitro* upregulation of PD-L1 expression in response to IFN-γ. **(A)** Left panel: Four replicates of A20 PD-L1 WT or PD-L1-deficient tumor cells were seeded in a 24-well plate at the rate of 5,000 cells per well, and cell counting was performed every day over a period of 6 days. The total number of cells (×10^6^) in culture is plotted at different time points. Middle panel: A representative experiment of two showing intracellular Ki-67 staining was used to measure the *in-vitro* fraction of dividing cells (Ki-67 positive) versus non-dividing cells (Ki-67 negative) in WT and PD-L1-deficient cell lines 1 day after seeding them at 3 × 10^5^ cells/ml. Right panel: A representative experiment of three illustrating cell death in cell culture measured at the exponential phase of cell growth by staining with propidium iodide. **(B)** A20 PD-L1 WT or PD-L1-deficient leukemia cells were left untreated or incubated for 24 h and 5 days with different concentrations of IFN-γ ranging from 0.5 to 2 μg/ml, and PD-L1 expression was monitored by flow cytometry. One experiment out of two with similar results. The mean fluorescence intensity (MFI) is indicated for each histogram.

The accumulation of living cells in cell culture results from the balance of cell division, cell survival, and cell death. We then performed *in-vitro* studies of tumor cell proliferation to evaluate whether loss of PD-L1 in A20 leukemia cells affected their overall survival or delayed its growth rate *in vitro*. An equal number of WT (A20-WT) or PD-L1-deficient cell line (A20-PD-L1 KO) was seeded in 24-well plates under the same culture conditions, and cell counting was monitored every day from day 1 to day 6. The results shown in **Figure 2A** (left panel) demonstrated a similar and parallel growth rate for both the A20 WT control and the A20 PD-L1-deficient cell lines.

During the course of the cell cycle, cells go through a sequence of phases starting in the G1 phase, continuing to the S and G2 phases and finishing in the M phase. Ki-67, a proliferating cell nuclear antigen, is a measure of the fraction of cells entering the cell cycle and mitosis, often used to evaluate the proliferating fraction versus the non-proliferating fraction within a tumor. The fraction of non-dividing cells was similar in A20 PD-L1-deficient cells (0.90%) compared to their WT counterparts (0.73%) in the exponential phase of cell growth, suggesting that deficiency in PD-L1 did not impact cell proliferation in A20 PD-L1 tumor cells (**Figure 2A**, middle panel). Cell death at the exponential phase of cell growth was negligible as assessed by propidium iodide intake and similar in both tumor cell lines (**Figure 2A**, right panel).

These data suggest that the indel mutations introduced into the PD-L1 gene led to a successful inactivation of protein expression but did not perturb the overall tumor cell growth and survival *in vitro.*

### Slight Upregulation of PD-L1 on A20 Tumor Cells in Response to Exposure of IFN-γ *In Vitro*

Most transplantable syngeneic tumor cell lines upregulate PD-L1 in response to IFN-γ to counterattack and evade cytolytic T cells through PD-1 co-inhibition of their functional activity (41). This emulates the behavior of naturally developed tumors *in vivo* that acquire adaptive mechanisms of resistance by augmenting PD-L1 expression, in response to IFN-γ released by antitumor CTLs (PD-L1 et al., 2018).

To prove that A20 leukemia cells behave just like other transplantable syngeneic tumor models, the PD-L1 WT and its counterpart PD-L1-deficient A20 tumor cells were exposed *in vitro* to IFN-γ (200 ng/ml for 24 h) or left untreated to determine whether PD-L1 expression was modulated in response to this cytokine. As seen in **Figure 2B**, the A20 PD-L1 WT cell line in response to IFN-γ augmented slightly the PD-L1 expression compared to the untreated control, but the increase in IFN-γ concentration did not lead to a concomitant increase in PD-L1 expression. A concentration as low as 200–500 ng was sufficient to achieve a modest upregulation of PD-L1 expression in A20 tumor cells. As expected, the PD-L1-deficient cell line expressed PD-L1 neither in resting conditions nor in response to the exposure to IFN-γ.

To sum up, the A20 transplantable leukemia tumor model, like many other syngeneic tumor cell lines, upregulates PD-L1 in response to IFN-γ.

### A20 Leukemia Cells Expressing PD-L1 Engrafted More Efficiently Than PD-L1 KO Tumor Cells in the Spleen but not in the Bone Marrow

**Figure 3:**
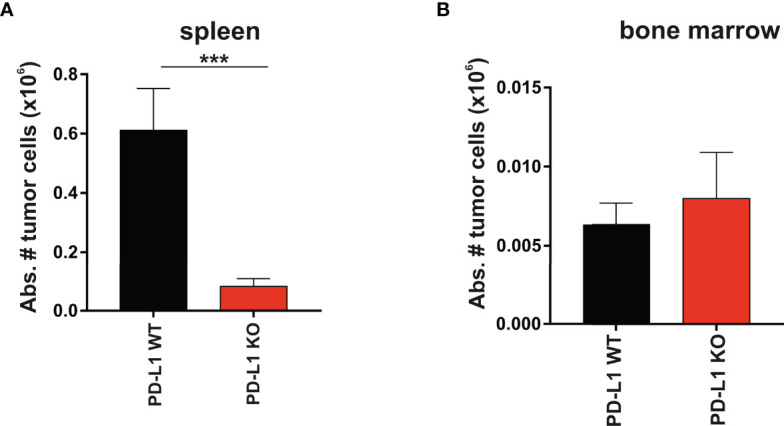
Superior engraftment of A20 PD-L1 WT tumor cells compared to A20 PD-L1-deficient tumor cells in the spleen of F1 recipients contrasted with the bone marrow resistance to tumor implantation of either tumor cell line. **(A)** Bar plot shows the absolute number of PD-L1 WT and PD-L1 KO leukemia cells in the spleen calculated at the time of the euthanasia (day 30 after tumor implantation). Tumor cells (K^b−^/K^d+^) were distinguished from non-tumor cells (host cells, K^b+^/K^d+^) by flow cytometry. **(B)** The absolute number of PD-L1 WT and PD-L1 KO leukemia cells in the bone marrow of F1 recipients was also monitored by flow cytometry at the time of the euthanasia. The absolute number of tumor cells was calculated from the cell suspension obtained after fluxing one tibia with culture medium. The plotted data represent the mean ± SEM from 10 to 15 mice per group. *p*-values were considered statistically significant according to the following criteria: ***p < 0.0005. Unpaired Student’s *t*-test was used to assess the statistical significance of the means. WT, wild type; KO, knockout.

The spleen and the bone marrow were the two hematopoietic compartments where tumor colonization was monitored. The abundance of immune cells responsible for hybrid resistance in primary and secondary lymphoid organs is probably tissue-specific. This may reflect the distinct tissue distribution and engraftment pattern of the tumor cells in the spleen versus the bone marrow. The PD-L1 expression on parental A20 WT tumor cells that colonized the spleen exhibited significant protection against rejection compared to A20 PD-L1-deficient tumor cells (**Figure 3A**, ***p < 0.0005). In contrast, the engraftment of parental A20 WT or A20 PD-L1 KO tumor cells in the bone marrow of F1 recipients did not follow the same pattern as that of the spleen. The implantation of tumor cells in the bone marrow of F1 recipients was residual and nearly undetectable regardless of whether PD-L1 was expressed or not. PD-L1 expression on tumor cells did not confer any advantage to the tumor for the colonization of this primary lymphoid organ, suggesting that PD-L1 expression on tumor cells may not provide sufficient protection against rejection by the host immune cells involved in hybrid resistance in this hematopoietic compartment (**Figure 3B**).

In summary, the data indicate that hybrid resistance to parental tumor engraftment follows a distinct rejection pattern in different hematopoietic compartments.

### The Increase in Liver Weight due to Clusters of Nodular Metastases Was Higher in F1 Recipients of A20 PD-L1 WT Tumor Cells Than in Those Receiving PD-L1-Deficient Tumor Cells

A20 leukemia cells express the CXCR4 chemokine receptor that guides them toward a chemokine gradient of CXCL12 (stromal cell-derived factor-1, SDF-1) actively produced by the biliary epithelium and bone marrow stromal cells (42, 43), which may account for the preferential metastatic behavior of A20 leukemia cells for these tissues.

**Figure 4:**
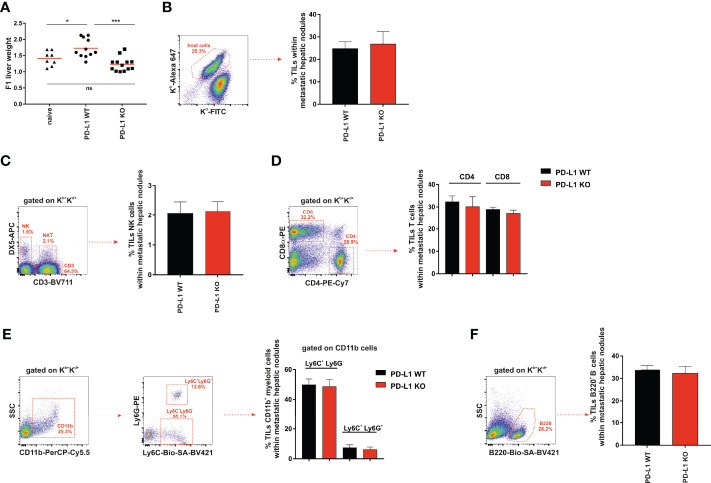
Significant increase in liver weight in F1 mice receiving A20 PD-L1 WT tumor cells compared to those implanted with A20 PD-L1 KO tumor cells. No substantial changes in tumor leukocyte infiltration in metastatic liver nodules arose from either A20 PD-L1 WT or PD-L1-deficient tumor cells. **(A)** The livers of non-treated F1 naive control mice (triangles) and F1 mice injected with either A20 PD-L1 WT (circles) or KO tumor cells (squares) were weighted at the time of the necropsy, 1 month after tumor injection. **(B)** The frequency of tumor-infiltrating leukocytes (TILs) in metastatic nodules of the liver was calculated and represented. The percentages of NK cells (CD3^−^/DX5^+^) **(C)**, CD4 and CD8 T cells **(D)**, CD11b^+^/Ly6C^+^/Ly6G^−^ (monocytes) and CD11b^+^/Ly6C^+^/Ly6G^+^ (granulocytes) **(E)**, and B cells (B220^+^) **(F)** were analyzed in metastatic liver nodules of F1 recipients engrafted with A20 PD-L1 WT or A20 PD-L1 KO tumor cells. Representative dot plots depicting the gating strategy and the subpopulations of each analysis are shown. The bar graph shows the mean ± SEM from 10 to 15 mice per group. *p*-values were considered statistically significant according to the following criteria: *p < 0.05; ***p < 0.0005. WT, wild type; KO, knockout. Unpaired Student’s *t*-test was used for the evaluation of the statistical significance of the means.

We compared PD-L1 WT and PD-L1 KO A20 tumor dissemination and the formation of metastases in the livers of F1 recipients 1 month after intravenous injection. The increase in liver weight due to metastatic nodules was higher in F1 recipients implanted with A20 PD-L1 WT leukemia cells than in those injected with A20 PD-L1 KO leukemia cells (**Figure 4A**, ***p < 0.0005) or naive F1 controls (**Figure 4A**, *p < 0.05). This indicates that PD-L1 expression on A20 leukemia cells confers a survival advantage to the tumor probably by inhibiting either the NK cell-mediated response or the CD8 T-cell-mediated response, the two immune cell contributors to hybrid resistance against the tumor.

We then assessed the frequency of tumor-infiltrating leukocytes inside the metastatic nodules of the liver, but no significant differences were found in the tumor arising from either A20 PD-L1 WT or KO leukemia cells (**Figure 4B**). We went on to analyze by flow cytometry the frequency of the different subpopulations of the host immune cells inside the metastatic nodules of the liver. No significant differences were found when the frequencies of NK cells (CD3^−^/DX5^+^) (**Figure 4C**), CD4 and CD8 T cells (**Figure 4D**), CD11b^+^/Ly6C^+^/Ly6G^−^ (monocytes) and CD11b^+^/Ly6C^+^/Ly6G^+^ (granulocytes) (**Figure 4E**), or B cells (B220^+^) (**Figure 4F**) were analyzed in F1 recipients of A20 PD-L1 WT or A20 PD-L1 KO tumor cells.

PD-L1 expression on parental A20 leukemia cells enhances tumor fitness and improves tumor survival in F1 recipients allowing efficient liver colonization by conferring them with a greater capacity to cope with the host resistance mechanisms of rejection.

### PD-L1 Expression on Parental Tumor Cells Does not Protect Against NK Cell-Mediated Hybrid Resistance

NK cells are particularly efficacious in the rejection of tumors lacking MHC class I expression or when MHC class I expression has been reduced (missing self-hypothesis) (1, 44). NK cells are also the main players involved in the rejection of parental bone marrow cells or parental hematopoietic tumors in F1 recipients (45–47).

**Figure 5:**
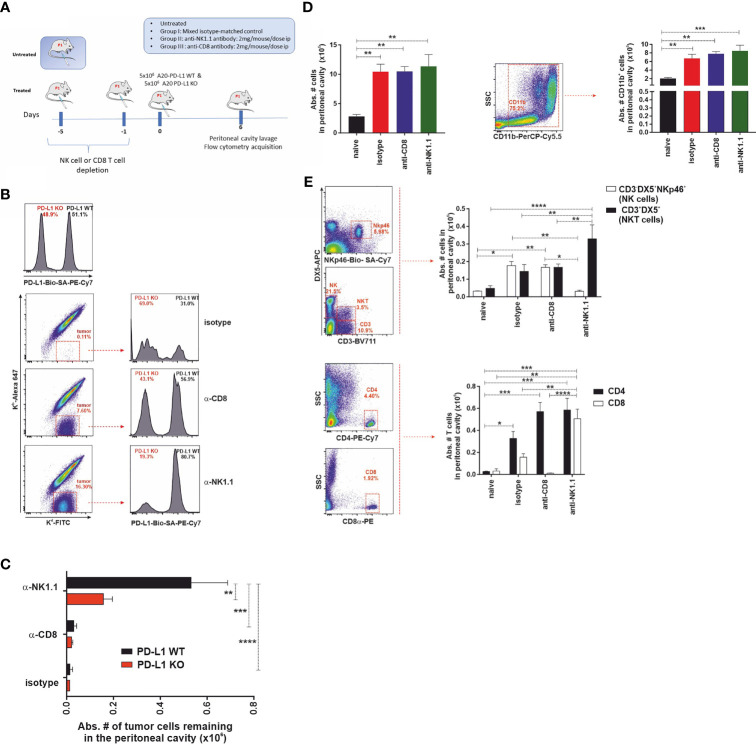
The expression of PD-L1 in A20 tumor cells confers protection against CD8 T-cell-mediated antitumor response but does not affect the NK cell-mediated component of hybrid resistance. **(A)** Experimental design to assess the contribution of host CD8 T cells and NK cells to the phenomenon of hybrid resistance in F1 mice receiving A20 PD-L1 WT and PD-L1-deficient leukemia cells into the peritoneal cavity. F1 recipient mice were treated with isotype control or depleting antibodies against CD8 T cells or NK cells at days −5 and −1 prior to tumor implantation at day 0. An equal number of A20 PD-L1 WT and A20 PD-L1 KO tumor cells were intraperitoneally injected and mice were euthanized on day 6 after tumor implantation. Then, the cellular composition of the peritoneal lavage was analyzed by flow cytometry distinguishing tumor cells (positive for MHC class I, K^d^) from non-tumor cells (host immune cells double-positive for MHC class I K^b^ and K^d^). **(B)** Top panel: A representative histogram example showing the ratio of the A20 PD-L1 WT/A20 PD-L1 KO tumor cell mix (ratio 1:1) after staining with anti-PD-L1 antibody and prior to the injection into the peritoneal cavity of F1 recipient mice. Bottom panel: A representative dot plot showing host cells (K^b+^/K^d+^) and tumor cells (K^b−^/K^d+^) of each experimental group (isotype-matched control and anti-CD8α- or anti-NK1.1-depleted F1 recipients are depicted). The percentage of A20 PD-L1 WT and A20 PD-L1 KO tumor cells remaining in the peritoneal cavity 6 days after tumor injection was calculated by excluding residual red cells and gating on K^b−^/K^d+^ tumor cells and the histogram plot shows PD-L1 staining to differentiate A20 PD-L1 WT from PD-L1 KO leukemia cells. **(C)** The absolute number of A20 PD-L1 WT and KO tumor cells remaining in the peritoneal cavity 6 days after their injection is depicted for isotype matched control and anti-CD8α or anti-NK1.1-depleted F1 recipients. **(D)** Tumor inoculation into the peritoneum attracts immune cells toward this location. Upper panel: The bar graph shows the absolute number of immune cells in the peritoneal cavity of tumor-bearing F1 mice compared to the normal number of immune cells in the peritoneal cavity of naive F1 mice. Representative dot plot depicting the gating strategy and the subpopulation of CD11b cells in the peritoneal cavity. The bar chart represents the absolute number of host CD11b myeloid cells recruited into the peritoneal cavity in response to tumor implantation compared with that of naive F1 mice. Middle panel: Representative dot plot depicting the gating strategy for NK cells and NKT cells in the peritoneal cavity. The bar chart represents the absolute number of NK cells (CD3^−^/DX5^+^/NKp46^+^ cells) and NKT cells (CD3^+^/DX5^+^) recruited into the peritoneal cavity of F1 mice in response to the inoculation of an equal number of A20 PD-L1 WT and KO tumor cells, 6 days after tumor injection. Lower panel: Representative dot plot depicting the gating strategy for CD4 T cells and CD8 T cells in the peritoneal cavity. The bar chart illustrates the absolute number of CD4 T cells and CD8 T cells in the peritoneal cavity of F1 mice co-injected with an equal number of A20 PD-L1 WT and KO tumor cells, 6 days after tumor injection. The plotted data represent the mean ± SEM from 5 to 8 mice per group. *p*-values were considered statistically significant according to the following criteria: *p < 0.05; **p < 0.005; ***p < 0.0005; ****, *p* < 0.00005. Student’s *t*-test and two-way ANOVA were used to assess the statistical significance of the means. WT, wild type; KO, knockout.

We hypothesized that if the PD-1 receptor were expressed in NK cells, as claimed by some authors, then one would expect that PD-L1 WT tumor cells would exhibit a resistance advantage over PD-L1 KO tumor cells and, consequently, they would be less vulnerable to NK cell-mediated rejection than PD-L1 KO tumor cells. To test that hypothesis, a short-term experimental strategy was designed to elucidate the relative contribution of cytotoxic cells (host NK cells or host CD8 T cells) to tumor clearance in the peritoneal cavity of F1 recipients (**Figure 5A**). Parental A20 WT and PD-L1-deficient tumor cells were co-injected in equal numbers (5 × 10^6^ of each cell type) in isotype control-treated, NK cell-depleted, or CD8 T-cell-depleted F1 recipient mice, and tumor survival was assessed 6 days after injection (**Figures 5B, C**).

We monitored the recruitment of leukocytes into the peritoneal cavity in response to co-injection of A20 PD-L1 WT and KO tumor cells, which mimics somehow a sterile inflammatory environment, promoted by tumor implantation and danger signals linked to damage-associated molecular patterns (DAMPs) detected by immune cells (48). A statistically significant increase in the total number of host immune cells in the peritoneal cavity of tumor-bearing F1 recipients was seen in response to the presence of tumor cells when compared to control naive F1 mice (**Figure 5D**, upper left, **p < 0.005). This increase in immune cells was mainly due to the recruitment of CD11b myeloid cells in tumor-bearing mice compared to naive F1 controls (**Figure 5D**, upper right, **p < 0.005).

The immune cell populations of the peritoneal cavity likely to participate in hybrid resistance were also monitored (CD3^−^/DX5^+^ NK cells, NKp46-positive subpopulation of NK cells and CD3^+^/DX5^+^ NKT cells) by flow cytometry in the different experimental groups. NK cell recruitment to the peritoneal cavity of tumor-bearing mice was significantly greater than in naive F1 mice (**Figure 5D**, lower left panel). Within the NK cell pool, the NKp46 cell subpopulation was the most vulnerable to NK cell depletion as this was significantly reduced in NK cell-depleted tumor-bearing F1 mice compared to the isotype control or the anti-CD8 T-cell-depleted group (**Figure 5D**, lower left panel). On the other hand, a significant increase of NKT cells was also observed in NK cell-depleted mice compared with the rest of the groups (**Figure 5D**, lower right panel). Regarding T cells, host CD4 T cells were significantly increased in tumor-bearing mice compared to naive F1 controls (**Figure 5D**, right lower panel). Host CD8 T cells also increased significantly in the NK cell-depleted group when compared to the rest of the experimental groups (**Figure 5D**, right lower panel).

In line with previous findings in the context of parental bone marrow transplantation into F1 recipients, depletion of the host NK cells was the major immune mechanism involved in hybrid resistance to parental A20 tumor cells (**Figure 5E**). Irrespective of whether PD-L1 was expressed or not on the cell membrane of the A20 leukemia cells, tumor cells were readily rejected with similar efficiency by NK cells in CD8 T-cell-depleted F1 mice (**Figure 5E**). A20 PD-L1 WT tumor cells survived significantly better in NK cell-depleted F1 recipients than in the anti-CD8 T-cell-depleted group or naive F1 controls (**Figure 5E**). Remarkably, apart from NK cells, the host CD8 T cells were also found to contribute to hybrid resistance, although to a much lower extent. Interestingly, the absolute number of A20 PD-L1 WT tumor cells remaining in the peritoneal cavity of F1 recipients was superior to that of A20 PD-L1 KO tumor cells in NK cell-depleted F1 recipients (**Figure 5E**). This means that PD-L1 expression on tumor cells protects them from CD8 T-cell rejection. These findings are in line with the current paradigm in cancer immunotherapy claiming that PD-L1 expression on tumor cells can effectively modulate CD8 T-cell-mediated antitumor responses.

In summary, PD-L1 expressed on tumor cells does not inhibit NK cell function ruling out the postulated claim that the PD-L1/PD-1 pathway contributes to modulating NK cell rejection of parental tumor cells.

### Neither Homeostatic nor Inflammatory Conditions Led to the Expression of the Co-Inhibitory Receptor PD-1 on Host NK Cells

**Figure 6:**
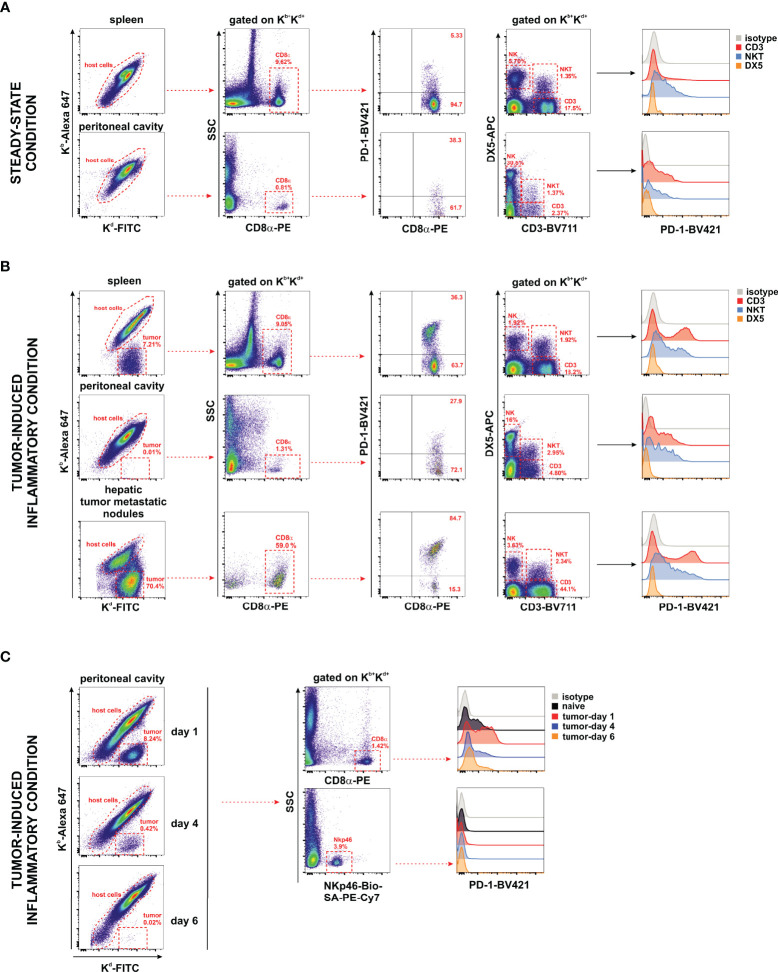
Undetectable PD-1 expression on NK cells under homeostatic or inflammatory conditions. Naive F1 mice **(A)** or A20 WT tumor-bearing F1 mice **(B)** were analyzed for the expression of PD-1 on NK cells (CD3^−^/DX5^+^), NKT cells (CD3^+^/DX5^+^), CD3 T cells, and CD8 T cells collected from the peritoneal cavity and spleen of naive mice (steady-state conditions) and from the spleen, peritoneal cavity, and liver metastases of tumor-bearing mice. Host immune cells co-expressing K^b+^/K^d+^ were differentiated from K^b−^/K^d+^ tumor cells in F1 mice injected with tumor cells. Representative dot plots of the analysis strategy in the spleen and metastatic nodules of the liver of F1 mice euthanized at day 30 after tumor implantation. One representative experiment out of three with similar results is depicted. **(C)** Time course expression of PD-1 on CD8 T cells and NK cells (NKp46^+^ cells) of the peritoneal cavity exposed to A20 WT tumor cells. Left panel: Representative dot plot showing host cells versus tumor cells remaining over the period of 6 days of follow-up depicting the kinetics of tumor rejection at days 1, 4, and 6 after tumor implantation. Right panel: Representative dot plot illustrating the gating strategy of CD8 T cells and NKp46^+^ NK cells for the analysis of PD-1 expression.

The expression of the PD-1 co-inhibitory receptor was monitored in F1 recipients under steady-state conditions (naive F1 mice, **Figure 6A**) and inflammatory conditions in the liver, spleen, metastatic nodules of the liver, and peritoneal cavity of A20 tumor-bearing F1 mice (**Figure 6B**). Whereas the expression of PD-1 is readily detectable on CD8 T cells and NKT cells, a complete lack of PD-1 expression was observed in NK cells irrespective of the tissue compartment analyzed (**Figures 6A, B**). PD-1 upregulates its expression upon CD8 T-cell and NKT cell activation in tumor-bearing mice in different hematopoietic compartments suggesting that A20 tumor cells are immunogenic and susceptible to be recognized by the immune system but are undetected in NK cells. The upregulation of PD-1 expression on CD8 T cells is dependent on the presence of the tumor in the peritoneal cavity and appears soon, declining later on gradually as tumor cells fade out due to the antitumor response (**Figure 6C**).

Our data support the hypothesis that in this mouse hematopoietic tumor model, the co-inhibitory receptor PD-1 is absent in NK cells suggesting that tumor cells bearing PD-L1 expression cannot directly co-inhibit NK cell function through PD-1.

## Discussion

Since the initial efforts involved in the generation of inbred strains of mice, researchers soon realized that tumor cell lines enjoy immune privilege features as they could engraft in histoincompatible hosts across some minor mismatch barriers while skin grafts were always consistently rejected. This suggests that tumors are endowed with the capacity to adapt and escape the antitumor response. Hematological tumors have even challenged the laws of transplantation formulated by Little, Gorer, and Snell (49, 50). Thus, parental skin grafts are accepted by F1 hybrids, whereas parental hematopoietic bone marrow cells, lymphoid cells, or tumor cells are eliminated by an NK cell-mediated mechanism of rejection (known as *hybrid resistance*) (6, 9, 51–55).

Today, it is universally accepted that tumors undergo genetic mutations that give rise to neoantigens that may become immunogenic and susceptible to recognition by the immune system. Tumors have evolved several direct and indirect mechanisms to evade recognition and resist the antitumor responses. Thus, for instance, tumor cells co-opt physiological regulatory mechanisms of tissues that have naturally evolved to prevent the development of immunopathology when they are exposed to long-lasting chronic inflammation. The upregulation of ligands for the co-inhibitory receptors, such as PD-L1, is one of the most relevant modulatory mechanisms to dampen inflammation and thus defend the living organism against the immune attack of cytotoxic T lymphocytes (15–17; PD-L1 et al., 2018). Accumulating evidence suggests that multiple immune evasion mechanisms may simultaneously operate in patients with advanced tumors. However, the contribution of each of these mechanisms to immune evasion and their temporal cross-regulation during tumor progression remain to be defined.

The A20 leukemia cell line was used as the hematopoietic tumor model because it lacks PD-1 expression *in vitro* or *in vivo*; therefore, trogocytosis (a phenomenon that permits immune cells to acquire relevant molecules from the cell surface of tumors or cells of the tumor microenvironment) is unlikely to occur and this scenario can be discarded. In addition to this intrinsic feature, A20 leukemia cells barely express CD80 allowing PD-L1 to interact in *trans* with PD-1 expressed on immune cells. Moreover, as shown for other transplantable tumor cell lines, the exposure to IFN-γ upregulates PD-L1, allowing the tumor to acquire a competitive advantage to co-inhibit the cytotoxic function of T cells (41).

Parental hematopoietic tumors engraft into F1 recipients as do hematopoietic bone marrow transplants due to their ability to overcome the barrier of hybrid resistance (6, 47, 51). In this scenario, parental tumor cells manage to escape NK cell-mediated attack and disseminate throughout the hematopoietic system. As in dysfunctional T cells, NK cells driven by the continuous presence of the tumor cells can also become functionally impaired (exhausted), allowing tumor cells to thrive and move to different locations, giving rise to metastases (56). Despite the fact that F1 recipients resist tumor engraftment at the initial phase after their implantation, tumors manage to survive. Tumor cells are selected for variants that escape the antitumor response or the antitumor response becomes exhausted and dysfunctional due to their inability to cope with the tumor burden accumulated during the course of tumor progression.

An intense controversy has been set around the role of NK cells in the field of tumor immunotherapy in the context of PD-1/PD-L1 blockade. It is an open question whether PD-L1/PD-1 blockade could enhance NK cell functional activity, as the expression of PD-1 in this immune cell population has been difficult to demonstrate. Despite the proponents’ claim that PD-1 can be detected in human NK cells of healthy individuals and in the context of different diseases (25, 25, 57–59), emerging evidence supports that the PD-1 receptor is either non-detectable or at best minimally present and restricted to activated NK cells within the tumor under inflammatory conditions in humans and mice (21, 23–25, 60). As opposed to those predominant tenets, others claim that PD-1 is completely absent on the cell surface of NK cells (22, 61). Our data support the notion that NK cells lack the PD-1 co-inhibitory receptor on their cell surface independently of their location (outside or inside the tumor) or their degree of activation (steady-state conditions or inflammatory conditions). This correlates with the finding that the absence of PD-L1 on A20 leukemia cells does not increase susceptibility to tumor rejection by NK cells compared to PD-L1 WT tumor cells while cytolytic response mediated by CD8 T cells was sensitive to expression of PD-L1 on tumor cells.

To reconcile our data with previous reports attributing a critical role to NK cells in the context of PD-L1/PD-1 immune checkpoint blockade and to account for the findings observed in a set of patients who responded to PD-L1/PD-1 blockade therapy despite bearing a PD-L1-negative tumor, two hypotheses have been put forward (62). One possible scenario is that PD-L1 expressed on NK cells would cross-regulate antitumor CD8 T-cell responses by inhibiting DC activation through PD-1 that would ultimately result in a reduced ability to support CD8 T-cell priming (63). Moreover, in some tumors, PD-L1 expression appears to be upregulated in NK cells, and antibodies against PD-L1 would enhance their function and revert exhaustion (64).

In most tumor models of hematopoietic origin, parental tumor cells can engraft in F1 recipients despite host hybrid resistance to their implantation, unless poly I:C is administered to enhance host NK cell cytotoxic function (46, 65). This innate resistance of the host depends largely on the age of the recipient F1 mice as the aging process declines the function of NK cells (66). Similarly, parental bone marrow transplantation into F1 recipients in the absence of pharmacological NK cell activation leads to successful engraftment and long-term multilineage donor chimerism in low-dose-irradiated (1–3 Gy) F1 recipients or even in non-irradiated recipients. The chimerism levels in F1 recipients were, however, lower than those achieved after transplantation of syngeneic bone marrow, supporting the idea that host NK cells of F1 recipients resist the engraftment of parental bone marrow cells (47, 52, 67). As expected and in agreement with these previous antecedents, the intravenous injection of parental A20 leukemia tumor cells into F1 recipients led to the systemic dissemination of tumor cells. Tumor cell distribution within hematopoietic and non-hematopoietic niches likely obeys a balance of preferential tropism and tissue-specific forces of resistance that in turn is the result of the relative composition and abundance of innate cells and CD8 T cells on those tissue compartments. This may account for the finding that F1 recipients are refractory to tumor implantation in the bone marrow regardless of PD-L1 expression. Neither PD-L1 WT A20 leukemia cells nor PD-L1 KO A20 leukemia cells were able to settle in great numbers in the bone marrow compartment of F1 recipients, although this hematopoietic site represents a niche for metastases in syngeneic Balb/c recipients. This was an unexpected finding as the bone marrow stromal niche is enriched with a chemokine gradient of CXCL12 (SDF-1) that may potentially attract A20 leukemia cells expressing the CXCR4 chemokine receptor (42, 43). The impossibility for the tumor to engraft successfully into the bone marrow of F1 recipients likely reflects the local hybrid resistance in this hematopoietic compartment, which is considered by many authors as a secondary lymphoid organ with immunological function of defense against foreign entities and not uniquely devoted to the maturation of immune cells (68). In this respect, a recent work has pointed out that bone marrow macrophages are important players in resisting the engraftment of syngeneic tumor cells and allogeneic bone marrow cells (18). Moreover, PD-L1/PD-1 interplay is a relevant pathway modulating the phagocytosis of tumor cells by a subset of macrophages expressing PD-1, extending the modulatory function of this co-inhibitory ligand to the regulation of phagocytic cells (18). The poor implantation of A20 PD-L1-deficient tumor cells compared with A20 PD-L1 WT cells in the spleen and liver may reflect their greater vulnerability to be eliminated by the host immune system.

To account for this differential behavior in tumor tropism and based on the data gathered from the A20 tumor model implanted into the peritoneal cavity, we observed that A20 PD-L1 WT tumor cells are protected to some extent from rejection by the host F1 CD8 T cells, likely due to PD-1-mediated inhibition of their cytotoxic function. As opposed to CD8 T cells, NK cell-mediated rejection of tumor cells was similarly efficient regardless of PD-L1 expression on tumor cells. This finding argues against the notion that PD-L1 expression on tumor cells inhibits NK cell function, and therefore, it is highly unlikely that PD-L1/PD-1 blockade would enhance NK cell-mediated tumor rejection or reverse NK cell functional impairment due to exhaustion.

CD8 T cells can also contribute to resist parental tumor engraftment in F1 recipients. This is likely due to the fact that parental tumor cells are seen as foreign entities in F1 recipients as they may express tumor-specific antigens that can be cross-presented by the host DCs to host CD8 T cells stimulating their cytotoxic response (69). Indeed, it is known that the A20 leukemia cell line bears a high tumor mutational burden being classified in the ranking of immunogenicity close to the MC38 colon carcinoma cell line. This antigenic tumor burden often correlates with a good response to treatment with anti-PD-L1 blocking antibody with the percentage of tumor growth inhibition of 71% (A20 leukemia) and 69% (MC38 tumor), respectively (69).

The initial purpose of this research was first to assess the role of PD-L1 expressed on tumor cells on NK cell function and second to determine the impact of PD-L1/PD-1 interaction in NK cell-mediated mechanisms of rejection. However, this experimental approach can also recreate a more general preclinical platform to study how to modulate the cytotoxic activity of NK cells and T cells against tumor cells and the reversal of their exhausted phenotype. Tumor cells in this mouse model evade the immune response and this could be reversed by therapeutic interventions aimed at blocking the co-inhibitory immune checkpoints to reinvigorate the functional activity of exhausted cytotoxic cells before they become fully dysfunctional.

## Data Availability Statement

The original contributions presented in the study are publicly available. These data can be found here: https://www.ncbi.nlm.nih.gov/ under the accession number OM975989.

## Ethics Statement

The animal study was reviewed and approved by the University of Alcala de Henares (authorization # OH-UAH-2016/015).

## Author Contributions

M-LR and J-IR-B designed the study, performed and analyzed the experiments, and wrote the manuscript. JP-S provided the reagents and shared his expertise in the field. All authors contributed to the final version of the manuscript.

## Funding

This work was supported by the Spanish Ministry of Science and Universities (Grant I+D+I # PID2019-103984-RB-I00, MCIN/AEI/10.13039/501100011033/), FEDER “Una manera de hacer Europa,” and the Department of Education of Castilla and Leon Regional Government (Grant # LE-006P20) to J-IR-B. It was also partially funded by the Spanish Network of Cancer Research, CIBERONC (Grant # CB16/12/00480).

## Conflict of Interest

The authors declare that the research was conducted in the absence of any commercial or financial relationships that could be construed as a potential conflict of interest.

## Publisher’s Note

All claims expressed in this article are solely those of the authors and do not necessarily represent those of their affiliated organizations, or those of the publisher, the editors and the reviewers. Any product that may be evaluated in this article, or claim that may be made by its manufacturer, is not guaranteed or endorsed by the publisher.

## Supplementary Material

Supplementary Figure 1 Generation of a PD-L1-deficient A20 tumor cell line using a CRISPR-Cas9 approach. **(A)** The indel mutation located at exon 3 of PD-L1 gene consisted of 5 bp insertion and 21 bp deletion (Genebank accession # OM975989). **(B)** PCR amplification of PD-L1 exon 3 from genomic DNA of A20 PD-L1 WT and A20 PD-L1 KO cell line. The expected band for PD-L1 exon 3 in A20 cell line was 342 bp whereas in PD-L1 deficient cell line was 326 bp. The following set of primers were used for PCR amplification: Primer forward exon3-F: 5’ CGTTTACTATCACGGCTCC 3’ and primer reverse exon 3-R: 5’ CATTGACTTTCAGCGTGA 3’. A 2.5% agarose gel was run to resolved the WT and KO PCR amplicons of PD-L1 exon 3. **(C)** Amino acid sequence alignment between exon 3 of A20-PD-L1 WT vs PD-L1 KO cells showing the frameshift mutation and the formation of several stop codons. The amino acid sequence alignment of the PD-L1 protein WT versus PD-L1 mutated protein was performed with Clustal Omega (http://www.ebi.ac.uk/tools/clustalo/). An asterisk displays identical amino acids indicating perfect alignment (*). Amino acid strong similarity or weak similarity are represented by (): or (.), respectively.

## Abbreviations

CD, Cluster of differentiation; NK, Natural killer; NKT, Natural killer T cells; TCR, T cell receptor; MHC, Major histocompatibility complex; WT, Wild type; KO, Knock-out; MFI, Mean fluorescence intensity; BMCs, Bone marrow cells; mAb, Monoclonal antibody; PI, Propidium Iodide; FCS, Fetal calf serum; SD, Standard deviation; SEM, Standard error of the mean; F1 (Balb/c × B6): Balb/c (female) x B6 (male) F1 hybrid; IFN-g, gamma interferon; PD-1, Programmed death 1; CRISPR, Clustered regularly interspaced short palindromic repeats; i.p., intraperitoneal; PBS, Phosphate buffer saline; ILC, Innate lymphoid cells; SFM, Serum free medium.
