# Supplementary figures and images for: Differential Engraftment of Parental A20 PD-L1 WT and PD-L1 KO Leukemia Cells in Semiallogeneic Recipients in the Context of PD-L1/PD-1 Interaction and NK Cell-Mediated Hybrid Resistance
Source: Front Immunol. 2022 Jun 20;13:887348. doi: 10.3389/fimmu.2022.887348 (PMC9251058; doi:10.3389/fimmu.2022.887348)

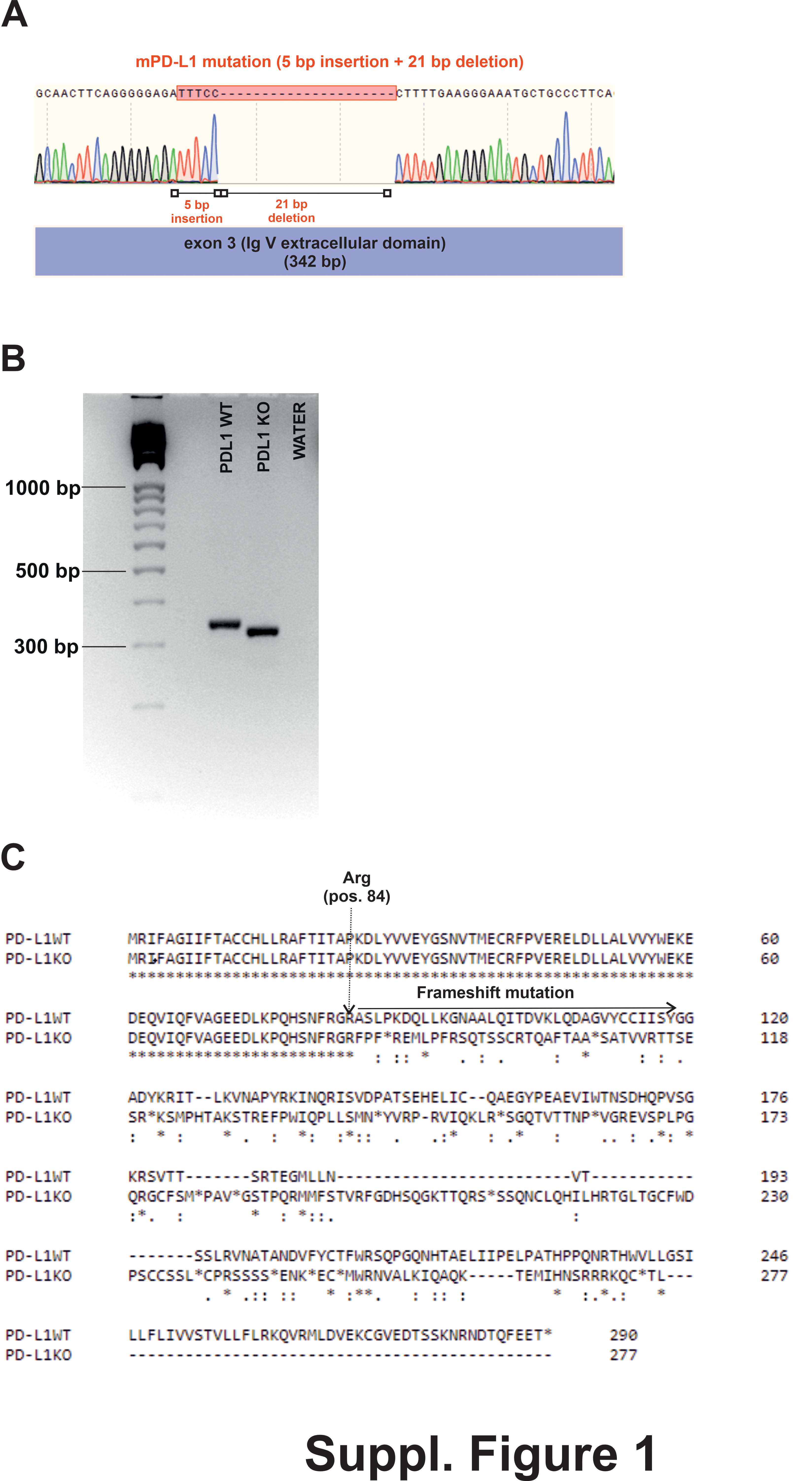

Supplement: Supplementary Figure 1 — Generation of a PD-L1-deficient A20 tumor cell line using a CRISPR-Cas9 approach. (A) The indel mutation located at exon 3 of PD-L1 gene consisted of 5 bp insertion and 21 bp deletion (Genebank accession # OM975989). (B) PCR amplification of PD-L1 exon 3 from genomic DNA of A20 PD-L1 WT and A20 PD-L1 KO cell line. The expected band for PD-L1 exon 3 in A20 cell line was 342 bp whereas in PD-L1 deficient cell line was 326 bp. The following set of primers were used for PCR amplification: Primer forward exon3-F: 5’ CGTTTACTATCACGGCTCC 3’ and primer reverse exon 3-R: 5’ CATTGACTTTCAGCGTGA 3’. A 2.5% agarose gel was run to resolved the WT and KO PCR amplicons of PD-L1 exon 3. (C) Amino acid sequence alignment between exon 3 of A20-PD-L1 WT vs PD-L1 KO cells showing the frameshift mutation and the formation of several stop codons. The amino acid sequence alignment of the PD-L1 protein WT versus PD-L1 mutated protein was performed with Clustal Omega (http://www.ebi.ac.uk/tools/clustalo/). An asterisk displays identical amino acids indicating perfect alignment (*). Amino acid strong similarity or weak similarity are represented by (): or (.), respectively. [file Image_1.tif]
